# The association between micronutrients and the SARS-CoV-2-specific antibodies in convalescent patients

**DOI:** 10.1007/s15010-022-01774-2

**Published:** 2022-02-21

**Authors:** Maryam Panahibakhsh, Faramarz Amiri, Taher Doroudi, Mostafa Sadeghi, Pirhossein Kolivand, Fatemeh Alipour, Ali Gorji

**Affiliations:** 1 Shefa Neuroscience Research Center, Khatam Alanbia Hospital, Tehran, Iran; 2 Department of Anesthesiology, Tehran University of Medical Sciences, Tehran, Iran; 3 Neuroscience Research Center, Mashhad University of Medical Sciences, Mashhad, Iran; 4 Present Address: Epilepsy Research Center, Westfälische Wilhelms-Universität, Münster, Germany; 5 Department of Neurosurgery, Westfälische Wilhelms-Universität, Münster, Germany; 6 Department of Neurology with Institute of Translational Neurology, Westfälische Wilhelms-Universität, Münster, Germany

**Keywords:** Healthcare workers, Pandemics, Viral infection, Immune system, Antibodies

## Abstract

**Background:**

Various micronutrients play key roles in the immune responses to viral infection, antibody synthesis, and susceptibility to infection. This study aimed to investigate the role of micronutrients on the immune responses following SARS-CoV-2 infection.

**Methods:**

To evaluate humoral immunity following SARS-CoV-2 infection, the levels of SARS-CoV-2-specific IgM and IgG, as well as the concentrations of different micronutrients, were determined in 36 convalescent COVID-19 patients 60 days after infection. Furthermore, the correlation between biochemical and hematological parameters, clinical features, and the changes in adiposity with SARS-CoV-2 antibodies was evaluated.

**Results:**

Serum IgM and IgG antibodies were detected in 38.8% and 83.3% of recovered patients after 60 days of COVID-19 infection, respectively. The values of SARS-CoV-2-specific IgG were negatively correlated with the number of the platelet. Moreover, the values of SARS-CoV-2-specific IgM were positively correlated with LDH and the vitamin B_12_ concentration. Furthermore, a gender-specific association of SARS-CoV-2-specific IgG and IgM with vitamins D as well as with B_9_ and zinc was observed. A significant negative correlation was observed between the values of IgG with vitamin D in male participants and a positive correlation was detected between IgG values and B_9_ in female participants. Moreover, IgM levels with serum zinc values in females were negatively correlated.

**Conclusion:**

Our study suggests the potential role of micronutrients in gender-specific humoral immunity following SARS-CoV-2 infection. Further studies are required with a greater sample of subjects to substantiate the validity and robustness of our findings.

## Introduction

The innate immune system provides the first line of defense against the sarbecovirus severe acute respiratory syndrome coronavirus type 2 (SARS-CoV-2). The immune system contributes to the virus clearance, inhibits virus replication, improves tissue repair, and activates long-lasting acquired immune responses [1, 2]. Antibody response is an essential element of protective immunity during infection with SARS-CoV-2 [3]. The first antibodies to be produced after the initial SARS-CoV-2 infection are immunoglobulin M (IgM) and IgA which can persist for about a month. IgG antibodies can be detected within the first 2–3 weeks of the onset of Coronavirus Disease 2019 (COVID-19), which are usually detectable for longer periods [4, 5]. Although previous investigations of antibody persistence after infection with Middle East respiratory syndrome coronavirus or severe acute respiratory syndrome coronavirus indicate that coronavirus-specific IgG is sustained for at least 1–2 years [6], long-lasting immunity against SARS-CoV-2 still needs to be determined [7]. It has been suggested that subjects with SARS-CoV-2 antibodies, particularly anti-spike or anti-nucleocapsid IgG, have a substantial immunity for approximately 6 months [8–10]. The values of anti-SARS-CoV-2 antibodies correlate with the duration and severity of symptoms but do not develop in all patients [11, 12].

It has long been known that the dietary intake of certain micronutrients significantly modulates antibody synthesis and regulates susceptibility to infection [13]. Accumulating evidence indicates that various micronutrients, such as vitamins A and D, promote the formation of germinal centers and enhance antigen-specific antibody production [14, 15]. Micronutrient deficiency is associated with a decrease in antibody production due to the dysfunction of various immune cells and alterations of immune homeostasis [16]. Deficiencies in various micronutrients, such as zinc [17], iron [18], vitamin B_6_ [19], vitamin B_12_ [20], vitamin A [21], and vitamin C [22] can lead to impaired antibody production. A significant relation between the values of some micronutrients, such as vitamin D, and the mortality and morbidity of SARS-CoV-2 infection has been reported [23]. The present study aimed to evaluate the potential role of various micronutrients in the antibody response to SARS-CoV-2 infection by determining micronutrient values in patients with COVID-19.

## Materials and methods

Thirty-six healthcare workers of Khatam Hospital, Tehran, Iran, who were infected with SARS-CoV-2, participated in this study. Infection with SARS-CoV-2 was confirmed by the detection of (i) viral RNA in the nasopharynx and pharyngeal swab specimens using real-time reverse transcription-polymerase chain reaction, and (ii) common patterns of COVID-19 pneumonia in thoracic computed tomography (CT)-scan. All subjects were treated with a combination of various drugs, including hydroxychloroquine, oseltamivir, azithromycin, ribavirin, and remdesivir. The study was approved by the Ethics Committee of Shefa Neuroscience Research Center, Tehran, Iran. Informed consent was obtained from all participants.

The values of IgG and IgM antibodies against the nucleocapsid protein (N) in serum samples were measured 60 days after the onset of symptoms using an enzyme-linked immunosorbent assay (ELISA) method. SARS-CoV-2 IgG ELISA assay and SARS-CoV-2 IgM capture ELISA assay were used for serological evaluation of SARS-CoV-2 infection. Samples were diluted with standard diluent (1/101, 100 μl/well) and transferred to polystyrene 96-well microtiter plates (Pishtaz Teb diagnostics) coated with inactivated SARS-CoV-2 antigen (37 °C for 30 min). The antigen-coated plates were washed five times with 10 mM phosphate-buffered saline (PBS, Gibco, Germany; pH 7.4) with 0.1% Tween-20. These wells were washed five times using 1 × PBS and Tween-20 (Thermo Fisher Scientific, Germany). Then, anti-human IgG horseradish peroxidase (Santa Cruz, Germany) was added (100 μl/well) and the plates were incubated for 30 min (37 °C). After incubation, the plates were washed and 100 μl of 3,3′,5,5′-tetramethylbenzidine (Thermo Fisher Scientific, Germany) substrate was added and incubated for 15 min. Sulphuric acid was added to stop the reaction. The absorbance values were measured at 450 nm using an ELISA reader. Positive and negative controls were prepared and included in the respective wells. IgG and IgM were considered reactive if index values were greater than 1.1. Iron, zinc, copper, and selenium were measured using enzymatic assays. Vitamin B_12_, vitamin D, and folate levels were measured using the ELISA method. Using high-performance liquid chromatography, the values of vitamin A, vitamin C, and vitamin E in serum were measured. Moreover, complete blood count (CBC), aspartate aminotransferase (AST), alanine aminotransferase (ALT), lactate dehydrogenase (LDH), and C-reactive protein (CRP) were assessed.

Furthermore, height, weight, body mass index (BMI), waist–hip ratio (WHR), soft lean mass (SLM), the mass of body fat (MBF), and muscle balance of all participants were measured using an X-contact body composition analyzer [24]. Clinical and demographic data, including age, gender, occupation, date of onset of symptoms, duration of the recovery period, history of close contact with a patient, place of hospitalization, place of quarantine, use of assistive devices, the start of the usual daily activity, oxygen saturation, use of oxygen during treatment, medications, rehabilitation after recovery, history of exercise in the last 6 months, and underlying diseases were collected.

### Statistical analysis

Data were analyzed using GraphPad Prism software version 6. Correlation analysis was conducted to identify variables related to IgG, IgM, and micronutrients. Pearson and Spearman correlation analysis was performed. Data are presented as mean ± S.E.M. A *P* value of less than 0.05 was considered to be statistically significant.

## Results

A total of 36 healthcare workers (15 females, 21 males; 29–61 years; mean age 43.2 ± 1.5 years) with a history of moderate (*n* = 7) or severe (*n* = 29) COVID-19 infection [25] were enrolled in this study. All participants were infected with SARS-CoV-2, which was confirmed by the positive RT-PCR tests and the typical CT features of COVID-19 pneumonia. The blood oxygen saturation of the subjects during the infection varied between 60 and 95%. Among the subjects, 28 (77.7%) did not have any chronic diseases, whereas other participants suffered from chronic obstructive pulmonary disease (*n* = 2), diabetes (*n* = 3), hypertension (*n* = 3), and cardiovascular diseases (n = 3). Furthermore, analyses of BMI data indicated that 16 subjects were overweight (between 25 and 30 kg/m^2^) and 9 participants were obese (< 30 kg/m^2^). The values of SLM in arms, legs, and trunk were higher than the average level in 26 subjects (7 females and 19 males). Only 8 subjects have reported regular daily physical activity during the 6 months prior to infection. CBC and serum biochemistry analyses revealed polycythemia (*n* = 3), anemia (*n* = 2), higher AST (*n* = 3) or ALT (*n* = 5), and increased (*n* = 3) or decreased (*n* = 1) LDH levels (Table 1).

**Table 1:** Summary of clinical and laboratory data

Comorbidities	
Hypertension	3 (8.3%)
Diabetes	3 (8.3%)
COPD	2 (5.6%)
Cardiovascular disease	3 (8.3%)
Body composition	
Weight	80.5 ± 2.4 (57.7–126.3)
BMI	27.9 ± 0.6 (21.8–33.9)
Over weight (between 25 and 30 kg/m^2^)	16 (44.4%)
Obese (< 30 kg/m^2^)	9 (25%)
SLM higher-than-average	26 (72.2%)
Laboratory examinations	
LDH (U/L)	174 ± 8.2 (42–354)
Platelet (10^*3^/μL)	232.9 ± 8.5 (103–332)
Iron (mg/dL)	82.5 ± 5.3 (31–186)
Zinc (μg/dL)	104.8 ± 1.7 (88–135)
Vitamin D (ng/mL)	31.8 ± 2.6 (6.3–86.9)
Vitamin A (μg/mL)	0.4 ± 0.03 (0.3–0.6)
Vitamin C (mg/dL)	0.7 ± 0.1 (0.2–1.2)
Vitamin B_12_ (pg/mL)	311.7 ± 42.3 (190–712)
Vitamin E (μg/mL)	11.2 ± 0.8 (8–17)
Vitamin B_9_ (ng/mL)	11 ± 1.4 (4.6–20)
Selenium (μg/dL)	67.6 ± 4.2 (27–97.2)
Copper (μg/dL)	92.3 ± 3 (81–112)
IgG (index)	11.3 ± 1.4 (0.07–22.2)
IgM (index)	1.7 ± 0.3 (0.05–8.5)

Data are presented as mean ± S.E.M. and numbers in parentheses are minimum and maximum values

*BMI* body mass index, *LDH* lactate dehydrogenase

The seropositive rate of SARS-CoV-2-specific IgM after 60 days of infection was 38.8% (*n* = 14). SARS-CoV-2-specific IgM was not detected in 22 subjects (10 females and 12 males). The rate of positive SARS-CoV-2-specific IgG was 83.3% (*n* = 30). SARS-CoV-2-specific IgG was not detected in 6 subjects (2 males and 4 females). Serum SARS-CoV-2-specific IgG antibodies were detected in all patients with severe COVID-19. Among all participants, low vitamin D values (< 30 ng/mL) and low vitamin C (< 0.6 mg/dL) levels were observed in 16 (5 females and 11 males) and 3 (2 females and 1 male) subjects, respectively. Furthermore, iron deficiency anemia was observed in one participant (< 30 mg/dL; Table 1).

**Fig. 1:**
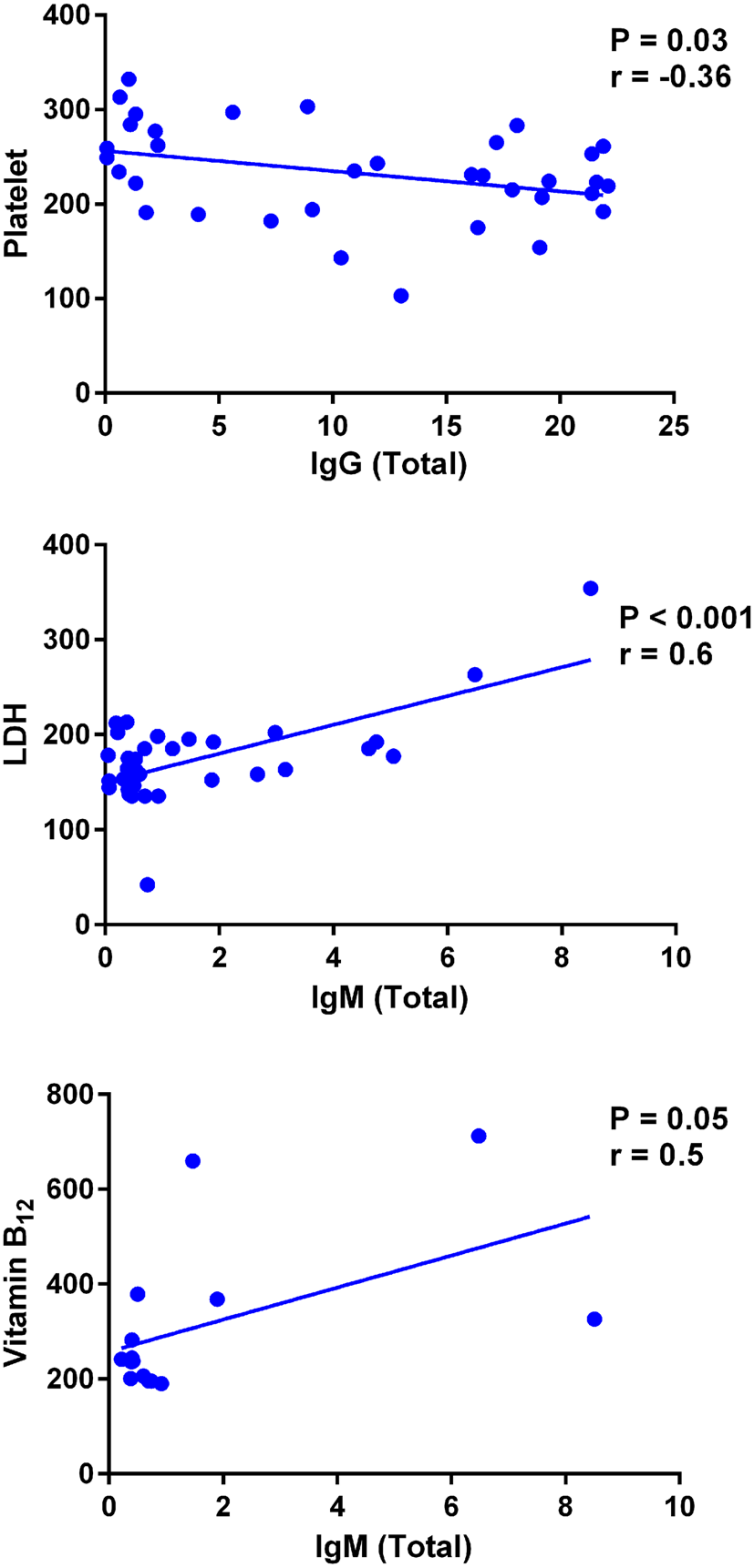
Correlation between the numbers of platelet, lactate dehydrogenase (LDH) concentrations, and vitamin B12 values with SARS-CoV-2 IgG or IgM antibodies in convalescent patients. Note the negative correlation between the numbers of platelet with SARS-CoV-2 IgG values, the positive correlation between LDH and SARS-CoV-2 IgM levels, and the positive correlation between vitamin B12 and SARS-CoV-2 IgM concentrations. The values were examined in subjects 60 days after infection with SARS-CoV-2

Correlation analysis between SARS-CoV-2 antibodies and different parameters of CBC has shown a significant negative correlation between the IgG values and the mean number of platelet (*r* = − 0.36; *P* = 0.03; Fig. 1). There was also a significant positive correlation between the IgM levels and LDH values (*r* = 0.6; *P* ≤ 0.001; Fig. 1). Furthermore, a positive correlation between the IgM levels and Vitamin B_12_ values (*r* = 0.5; *P* = 0.05; Fig. 1) was observed. There was no correlation between the values of SARS-CoV-2 antibodies with other micronutrients, the severity of disease, age, length of hospitalization, comorbidities, BMI, WHR, soft SLM, MBF, and muscle balance.

**Fig. 2:**
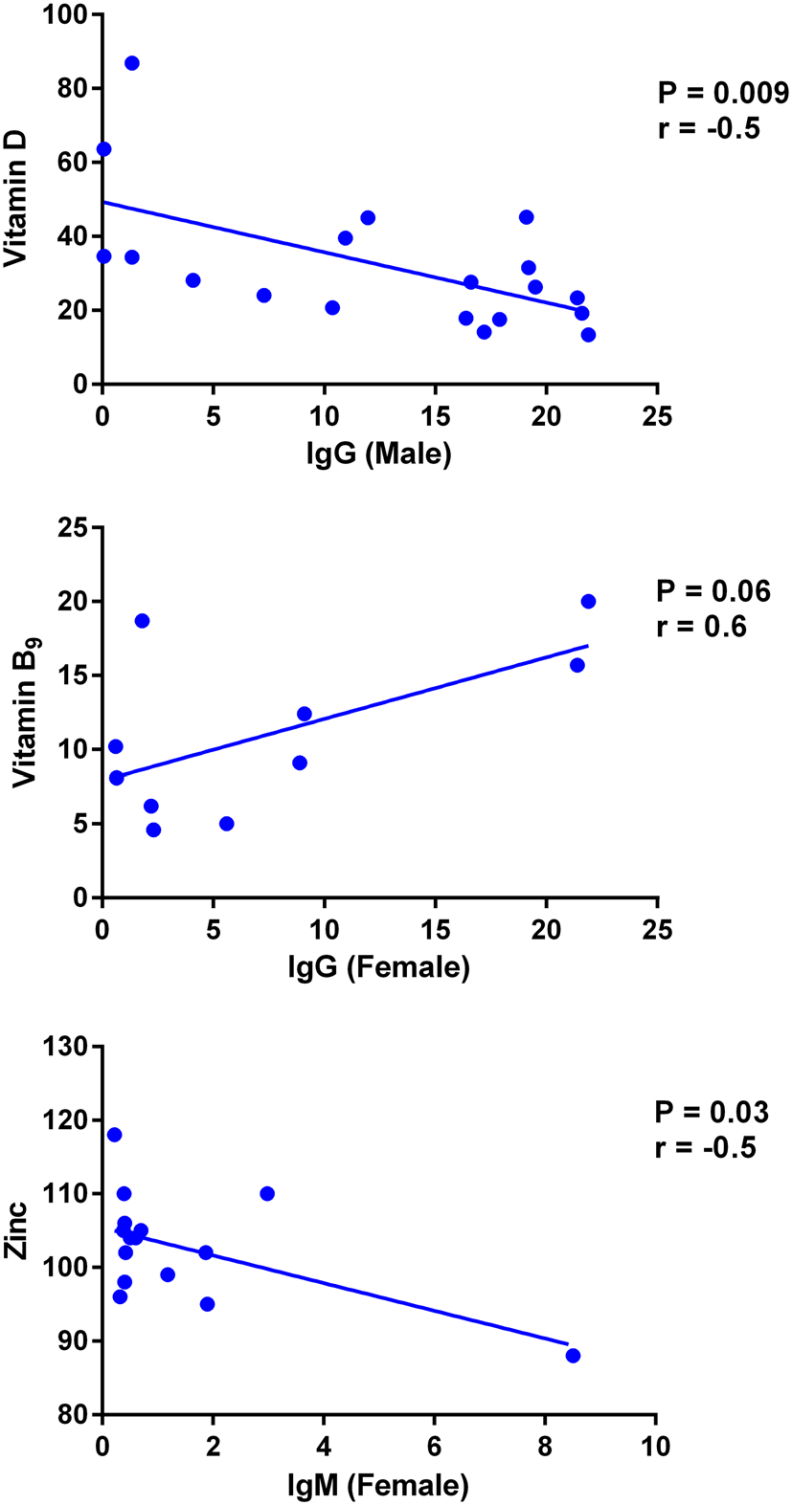
Correlation between the values of vitamins D and B_9_ as well as zinc with SARS-CoV-2 IgG or IgM antibodies in convalescent patients. Note the negative correlation between the levels of vitamin D with SARS-CoV-2 IgG values in male subjects, the positive correlation between vitamin B_9_ and SARS-CoV-2 IgG concentrations in female subjects, and the negative correlation between zinc and SARS-CoV-2 IgM levels in female subjects. The values were measured 60 days after infection with SARS-CoV-2 in 36 subjects

Micronutrients act differently on the immune responses of males and females [26]. Therefore, we divided the subjects into the male and female groups and examined the potential correlations between micronutrients and the production of antibodies in these two groups. A significant negative correlation was observed between serum IgG and vitamin D values in male participants (*r* = − 0.5; *P* = 0.009; Fig. 2). Furthermore, there was a positive correlation between IgG levels and B_9_ concentrations in female subjects (*r* = 0.6; *P* = 0.06; Fig. 2). Moreover, the levels of IgM have a significant negative correlation with serum zinc concentrations in female subjects (*r* = − 0.5; *P* = 0.03; Fig. 2).

## Discussion

The dynamics of SARS-CoV-2-specific antibodies in recovered patients have crucial implications for the diagnosis and treatment of COVID-19. Identification of the factors that contribute to the longevity of immune responses in patients with COVID-19 is essential for determining the risk of reinfection in previously exposed subjects [27]. In keeping with previous studies, our data indicate that serum IgM and IgG antibodies were detected in 38.8% and 83.3% of convalescent patients, respectively, after 60 days of COVID-19 infection [28]. Moreover, the present data suggest correlations between LDH, vitamin B_12_, and the number of platelets with SARS-CoV-2-specific antibodies. Furthermore, our results indicate a gender-specific association of SARS-CoV-2-specific IgG and IgM with vitamin D, vitamin B_9_, and zinc.

LDH, a membrane-associated enzyme, is released into the extracellular micromilieu during inflammation [29]. Several investigations have shown that a marked increase in LDH values was associated with poor outcomes in patients with COVID-19 [30]. In keeping with our results, a previous study has revealed that the serum IgM value was positively correlated with LDH concentration in patients with COVID-19 [31]. Besides IgM, the IgG responses against the S1 protein of SARS-CoV-2 were positively correlated with the enhancement of LDH in convalescent patients [32]. A positive correlation between LDH concentration and the IgM value has also been reported in other viral infections, such as Epstein–Barr–Virus [33]. In addition to enzymatic activity, LDH has an immunoglobulin production-stimulating factor domain [34] that modulates the production of various immunoglobulins, such as IgM [35]. Alterations in the mean number of platelets have been reported to be correlated with morbidity and mortality in patients with COVID-19 [36]. SARS-CoV-2 infection can lead to mild thrombocytopenia due to increased platelet consumption [37] and consequently to an increase in platelet production, activation, and aggregation [38]. Platelets play a modulatory role in the adaptive immune responses through the activation of peripheral blood B lymphocytes and the enhancement of immunoglobulin production [39]. Platelet–virus interplay regulates the host response to viral infection, which plays a crucial role in immune system function and illness outcomes [40]. Our data suggest that platelets may contribute to the production of SARS-CoV-2-specific antibodies.

Epidemiological studies revealed gender differences in the incidence, morbidity, and mortality of coronavirus infections, including COVID-19 [41, 42], which can be attributed to the diversity of the nature and strength of immune responses in women and men [43]. Both genetic and hormonal factors are implicated in stronger antibody responses, greater immunoglobulin values, and higher numbers of B lymphocytes in women than men [44, 45]. The immunomodulatory effects of estrogen, as well as the immune-suppressing effects of testosterone, can lead to stronger immune responses in females [46]. Females have greater antibody responses to various vaccines, such as hepatitis A and B, smallpox, influenza, and rabies [47, 48]. Micronutrients regulate the development and function of the immune system differently in women and men [49]. A significant reduction of serum vitamin A level was observed in females after measles and measles–mumps–rubella vaccines [50]. Vitamin A given with the measles vaccine decreased leukocyte subsets in males and enhanced the production of interferon-γ, as well as lymphocyte, monocyte, and basophil cell counts, in females [51]. Vitamins B, C, and E supplementation during pregnancy and after delivery exerted stronger beneficial effects on the reduction of low birth weight and mortality among girls born to HIV-infected women [52]. Vitamin D produces greater inhibition of pro-inflammatory cytokines and a higher enhancement of anti-inflammatory cytokines in females suffering from inflammatory disorders [53]. The synergy between estrogen and vitamin D may contribute to the gender differences in clinical outcomes of patients with COVID-19. [54]. Micronutrient supplementation in early gestation has gender-differential effects on the immune system in offspring, particularly on an epigenetic level [55]. Vitamin D modulates the activity of natural killer cells in a gender-dependent manner [56], presumably via the molecular interaction between estrogen and vitamin D [57].

Accumulating evidence indicates the potential role of these micronutrients in the humoral immune response to SARS-CoV-2 infection [16]. We observed a positive correlation between the B12 and IgM values in convalescent patients. Vitamin B_12_ regulates lymphocyte function via the enhancement of T-cell activities and modulates immunoglobulin synthesis [58, 59]. A potential link between high plasma values of vitamin B12 and enhanced risk of mortality in patients with COVID-19 has been reported [60]. Furthermore, our study revealed a negative correlation between the values of vitamin D with the production of IgM in male patients. Vitamin D reduces the numbers of memory B lymphocytes and inhibits the generation of plasma cells, with the consequent reduction in the secretion of various antibodies, including IgG and IgM [61]. Serum 25-hydroxyvitamin D values are lower in hospitalized patients with COVID-19 compared with population controls [62] and subjects with vitamin D deficiency have a greater chance of getting severe SARS-CoV-2 infection [63, 64]. A case–control investigation of hospitalized patients in Iran revealed a significant negative correlation between the serum vitamin D values and developing SARS-CoV-2 infection [65]. Vitamin B_9_ is implicated in the proliferative responses of lymphocytes and antibody synthesis [66]. Our study indicates a positive correlation between B9 and IgG levels in female patients. Decreased folate value is highly prevalent in patients hospitalized with COVID-19 infection [67]. Zinc plays a crucial role in antibody production via the alterations of the function and number of various immune cells in inflammatory conditions [17]. IgM values in our patients have shown a negative correlation with serum zinc levels in females infected with SARS-CoV-2. Zinc augmented the suppressive effects of NF-κB on angiotensin-converting enzyme 2, a major receptor for SARS-CoV-2 infection [68], in a human lung cell line [69]. A study on hospitalized patients with COVID-19 in Iran has suggested that serum values of vitamin D, vitamin B12, and zinc at the time of admission negatively affect the clinical outcomes [70].

The present study has some limitations. Our investigation was a single-center study with a small sample size of healthcare workers. However, our data emphasize the potential role of micronutrients in humoral immune responses to SARS-CoV-2 and its accuracy can be confirmed in a larger randomized controlled trial with an appropriate control group. Furthermore, all participants in our study were healthcare workers, which may not represent the entire patient population. It should be noted that the seroprevalence of SARS-CoV-2-specific IgG and IgM antibodies in health workers was not significantly different from the general Iranian population [71]. Socioeconomic factors are not associated with seropositivity in the general population [72]. However, socioeconomic diversity is associated with differences in intake and values of certain micronutrients [73]. The participants of our study were all health workers and possess the same socioeconomic status. Socioeconomic status should be considered as an important factor in future studies.

## Conclusion

Altogether, our data suggest the potential role of some micronutrients in the post-infection immunity of COVID-19 in a limited number of cases. This may provide insight into the mechanisms implicated in the interaction between the SARS-CoV-2 infection and host immune response.

## Data Availability

The data are available upon reasonable request.

## Abbreviations

ALT: Alanine aminotransferase
AST: Aspartate aminotransferase
BMI: Body mass index
CBC: Complete blood count
CRP: C-reactive protein
COVID-19: Coronavirus Disease 2019
CT: Computed tomography
LDH: Lactate dehydrogenase
SARS-CoV-2: Severe acute respiratory syndrome coronavirus 2
SLM: Soft lean mass
MBF: The mass of body fat
WHR: Waist–hip ratio
